# Dynamic linkage of COVID-19 test results between Public Health England’s Second Generation Surveillance System and UK Biobank

**DOI:** 10.1099/mgen.0.000397

**Published:** 2020-06-18

**Authors:** Jacob Armstrong, Justine K. Rudkin, Naomi Allen, Derrick W. Crook, Daniel J. Wilson, David H. Wyllie, Anne Marie O’Connell

**Affiliations:** ^1^​ Big Data Institute, Nuffield Department of Population Health, Li Ka Shing Centre for Health Information and Discovery, Old Road Campus, University of Oxford, Oxford OX3 7LF, UK; ^2^​ UK Biobank, 1–2 Spectrum Way, Adswood, Stockport SK3 0SA, UK; ^3^​ Nuffield Department of Medicine, Experimental Medicine Division, University of Oxford, John Radcliffe Hospital, Oxford OX3 9DU, UK; ^4^​ Field Service, Public Health England, Addenbrooke’s Hospital, Cambridge CB2 0QQ, UK; ^5^​ Data and Analytical Services, National Infection Service, Public Health England, London NW9 5EQ, UK

**Keywords:** UK Biobank, Public Health England, database linkage, COVID-19, SARS-CoV-2, bugbank

## Abstract

UK Biobank (UKB) is an international health resource enabling research into the genetic and lifestyle determinants of common diseases of middle and older age. It comprises 500 000 participants. Public Health England’s Second Generation Surveillance System is a centralized microbiology database covering English clinical diagnostics laboratories that provides national surveillance of legally notifiable infections, bacterial isolations and antimicrobial resistance. We previously developed secure, pseudonymized, individual-level linkage of these systems. In this study, we implemented rapid dynamic linkage, which allows us to provide a regular feed of new COVID-19 (SARS-CoV-2) test results to UKB to facilitate rapid and urgent research into the epidemiological and human genetic risk factors for severe infection in the cohort. Here, we have characterized the first 1352 cases of COVID-19 in UKB participants, of whom 895 met our working definition of severe COVID-19 as inpatients hospitalized on or after 16 March 2020. We found that the incidence of severe COVID-19 among UKB cases was 27.4 % lower than the general population in England, although this difference varied significantly by age and sex. The total number of UKB cases could be estimated as 0.6 % of the publicly announced number of cases in England. We considered how increasing case numbers will affect the power of genome-wide association studies. This new dynamic linkage system has further potential to facilitate the investigation of other infections and the prospective collection of microbiological cultures to create a microbiological biobank (bugbank) for studying the interaction of environment, human and microbial genetics on infection in the UKB cohort.

## Data Summary

The code written for database linkage in this study is internal to Public Health England (PHE) systems, and will not be released publicly. The data provided by the PHE system will be incorporated into the UK Biobank (UKB) database and released through the usual governance processes. To access UKB data, researchers must register and submit a research application (https://www.ukbiobank.ac.uk/register-apply). Registration is open to all bona fide researchers for all types of health-related research that is in the public interest. The registration and application process ensures researchers and projects meet UKB’s obligations to its participants and funders.

**Significance as a BioResource to the community**

Infections are a major source of human disease around the world, both during outbreaks such as the ongoing COVID-19 pandemic, and in ordinary times. Scientific research provides the foundation of new knowledge about the risks and consequences of infection. This research can contribute to delivering new drugs and vaccines and to public-health policy. In this article, we report our contribution to facilitating research into the risk factors for severe COVID-19 and other infectious diseases by integrating information between two valuable resources: the UK Biobank (UKB) and a Public Health England national microbiology database. UKB involves citizens who have provided consent for their de-identified data to be accessed by approved researchers worldwide to perform health research that is in the public interest. Beginning in 2006, the study recruited men and women aged 40–69 years across the UK, and collected a vast array of lifestyle data, physical measures and biological samples (for genomic and other assays to be performed). These data, together with long-term linkage to their electronic medical records, provide an unprecedented resource to understand the epidemiology of diseases of middle and older age. In this article, we report a new computerized system that provides daily linkage of participants with their microbiological test results, with the aim of providing data about COVID-19 and other infections in the UKB cohort.

## Introduction

As of 19 May 2020, the coronavirus SARS-CoV-2 that causes the severe acute respiratory syndrome COVID-19 was reported to have infected over 5 million people and killed over 320 000 people around the world [1, 2]. Better understanding of this novel pathogen is urgently needed to help guide the improvement of treatment and prevention. Large cohort studies such as the UK Biobank (UKB), which have gathered detailed epidemiological, medical and genetic records of hundreds of thousands of people, offer the opportunity to uncover risk factors for COVID-19, including the molecular genetic pathways underlying severe disease.

UKB is a longitudinal prospective cohort study that aims to investigate the causes, treatment and prevention of many common diseases of middle and older age [3]. The cohort is a particularly appropriate focus for the study of COVID-19, because incidence of this severe disease increases with age [1, 4–10]. The UKB cohort comprises around 500 000 men and women from the UK who were aged 40–69 years when they were recruited in 2006–2010; in England, 427 000 individuals were still being followed up at the end of 2019. Participants attended assessment centres, provided detailed information on lifestyle and medical history, underwent a range of physical measures and provided biological samples for future assays. They also provided consent for UKB to follow their health over the longer term by linking to their health-related records. Research scientists around the world can register and apply for access to UKB data, allowing them to study lifestyle, environmental and human genetic risk factors for disease (https://www.ukbiobank.ac.uk).

Studies of infection within UKB mainly rely on identifying infection events among participants from electronic medical records. To date, this predominantly comes from hospital inpatient admissions, including the hospital episode statistics (HES), which contain diagnoses assigned by professional coding teams post-discharge based on medical records. Acute diagnoses, and those of underlying conditions, are codified using the ICD-10 (International Classification of Diseases) system. Emergency ICD-10 codes for COVID-19 have been assigned (U07.1 COVID-19, virus identified; U07.2 COVID-19, virus not identified; https://www.who.int/classifications/icd/COVID-19-coding-icd10.pdf). However, there are limitations to these data for studying infection, as coding occurs in local National Health Service (NHS) Hospital Trusts, with subsequent central collation by the NHS and periodic (currently monthly) incorporation of summaries into UKB. Other limitations of HES for studying infection include incomplete or insensitive microbiological testing, and difficulty in syndromic diagnosis, especially in the elderly [11], where infection can exacerbate pre-existing conditions, so that not all causes of infection-related hospitalization are necessarily recorded as such. Moreover, infection diagnosis, and its coding, is often imprecise: for example, ICD-10 permits broad non-specific categories to be recorded such as A41.9 ‘Septicaemia, unspecified’, of which there were 2660 cases among UKB participants by 2017.

The Public Health England (PHE) microbiology database SGSS (Second Generation Surveillance System) offers advantages over HES data for the ascertainment of infection in UKB participants, because it provides more granular and highly specific diagnosis of microbiological confirmed infection, including both COVID-19 and infections caused by micro-organisms with antimicrobial resistance (AMR). Of note, it only allows identification of microbiologically confirmed disease; in the case of COVID-19, other databases with a more clinical focus also exist, such as the PHE COVID-19 Hospitalization in England Surveillance System (CHESS) and Intensive Care National Audit and Research Centre (ICNARC) databases containing individual patient data on critically ill patients in intensive care units. The SGSS database provides coverage of English UKB participants, who make up 89 % of the cohort based on residence at the time of recruitment. For these reasons, we previously developed secure, pseudonymized, individual-level linkage between SGSS and UKB with a view to providing data feeds periodically, e.g. annually, as with other data sources like cancer registries [12].

The NHS and PHE have put in place microbiological testing for SARS-CoV-2. As of 14 May 2020, 111 laboratories had reported SARS-CoV-2 nucleic acid detection results to SGSS, of which 108 had reported positives and 101 had reported negatives; over 228 000 positive and 580 000 negative tests had been reported. As well as playing a critical role in patient diagnosis, these data are important to enable research into the epidemiological and genetic determinants of severe COVID-19. In this paper, we report the development of a dynamic linkage system that identifies new records in SGSS from UKB participants on a daily basis, and feeds those results back to UKB weekly.

We originally developed this system as a pilot study to determine the feasibility of prospective microbiological culture collection from UKB participants to create a microbiological biobank (bugbank) for joint studies of epidemiological, human genetic and pathogen genomic risk factors for infection. In light of the COVID-19 pandemic, we have repurposed the system to provide near-to-real-time data on SARS-CoV-2 positive and negative test results for UKB participants. Here, we characterize the first 1352 identified cases in the cohort and compare their demographic characteristics to the rest of the UKB cohort and to other cases in England.

## Methods and Results

### Dynamic data linkage

We established a dedicated server at PHE Colindale to manage dynamic linkage between UKB and SGSS. All NHS microbiological laboratories in England provide data to SGSS each working day. SGSS consumes two data feeds, performing quality-control checks and applying mappings between terms used by individual laboratories to produce a standardized dataset. The AMR feed contains data from all microbiological cultures on which AMR testing was performed. The communicable disease report (CDR) feed contains mandatory reporting of a narrow range of pathogens of particular public-health importance, including SARS-CoV-2. Our algorithms link to both the AMR feed for the prospective micro-organism retrieval pilot study and the CDR feed for the COVID-19 rapid response project.

There are specific challenges that arise when frequently linking data between the large SGSS and UKB participant databases. These challenges pertain to the computational demands of dealing with high-volume, high-frequency queries. Building on our previous static linkage approach [12], we developed a speed optimized algorithm [13] to implement incremental daily linkage of the circa 200 000 records fed into SGSS each day and to identify those belonging to UKB participants.

**Fig. 1.**
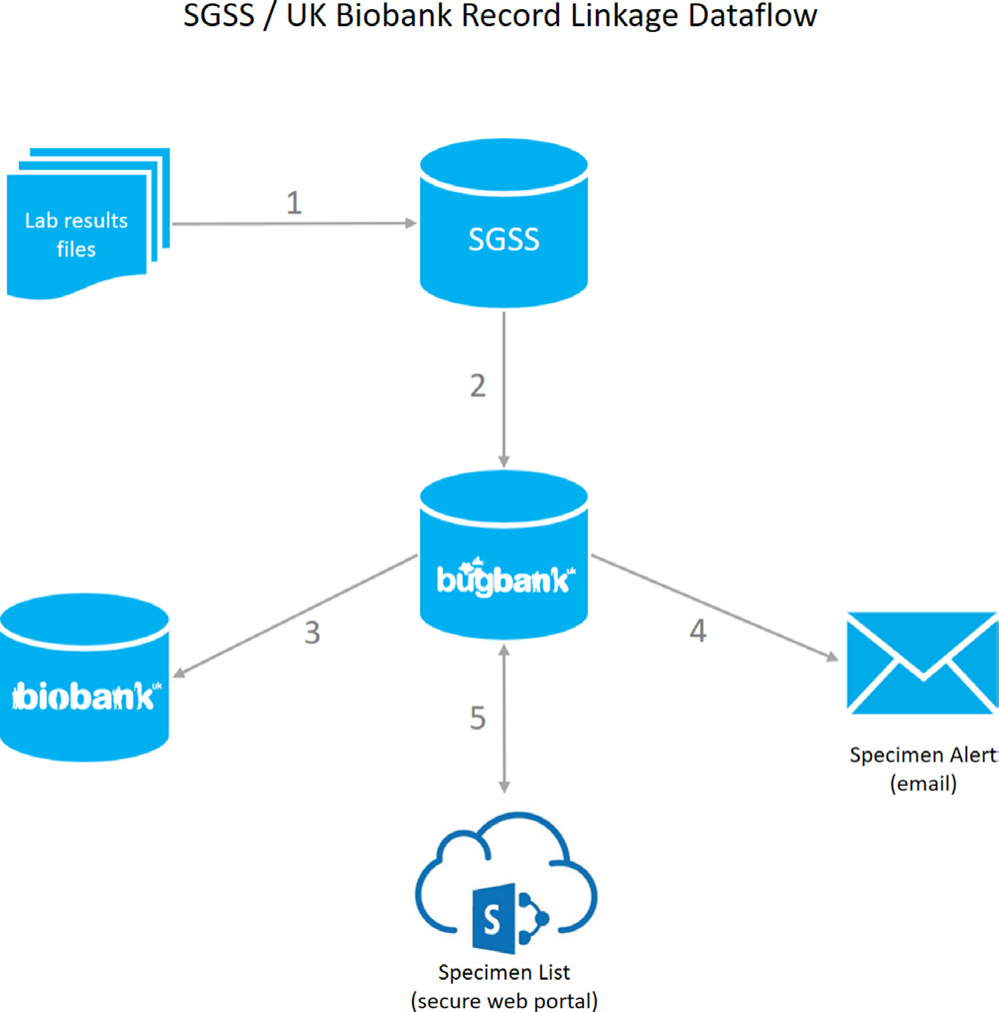
Information flow for identifying infection events among UKB participants in SGSS and issuing laboratory alerts to retrieve those micro-organisms. (1) A laboratory results file is received by SGSS from an NHS or PHE laboratory. (2) Hourly, an agent checks SGSS for new UKB infection events, and adds any to a separate database in PHE (Bugbank). The agent copies specimen and AMR susceptibility records. (3) Periodically, an extract of the data is transferred securely to UKB for incorporation into their system. (4) Daily, the agent sends an email alert to each active NHS or PHE laboratory. The email contains minimal information necessary for the laboratory to retrieve micro-organisms from UKB participant infections. (5) A secure SharePoint site provides a front end to view each laboratory’s specimens in PHE’s Bugbank database and records whether each micro-organism has been recovered, is missing or the record veracity has been questioned by the laboratory.

The key steps in our system, summarized in Fig. 1, are as follows.

- (1) An agent runs persistently on a server at PHE Colindale hosting SGSS, receiving daily updates from NHS/PHE laboratories across the country.
- (2) Periodically, the agent updates a database held at PHE Colindale with any new records from SGSS that it matches with UKB participants. The record matching procedure uses computerized pseudonymization (OpenPseudonymiser; www.openpseudonymiser.org) to maintain privacy and prevent inadvertent disclosure of patient identifiers, as previously described [12].

- (3) Periodically, an extract of the data is transferred to UKB for ingestion into their systems.

To enable a prospective culture collection feasibility study, the further steps undertaken are as follows.

**Fig. 2.**
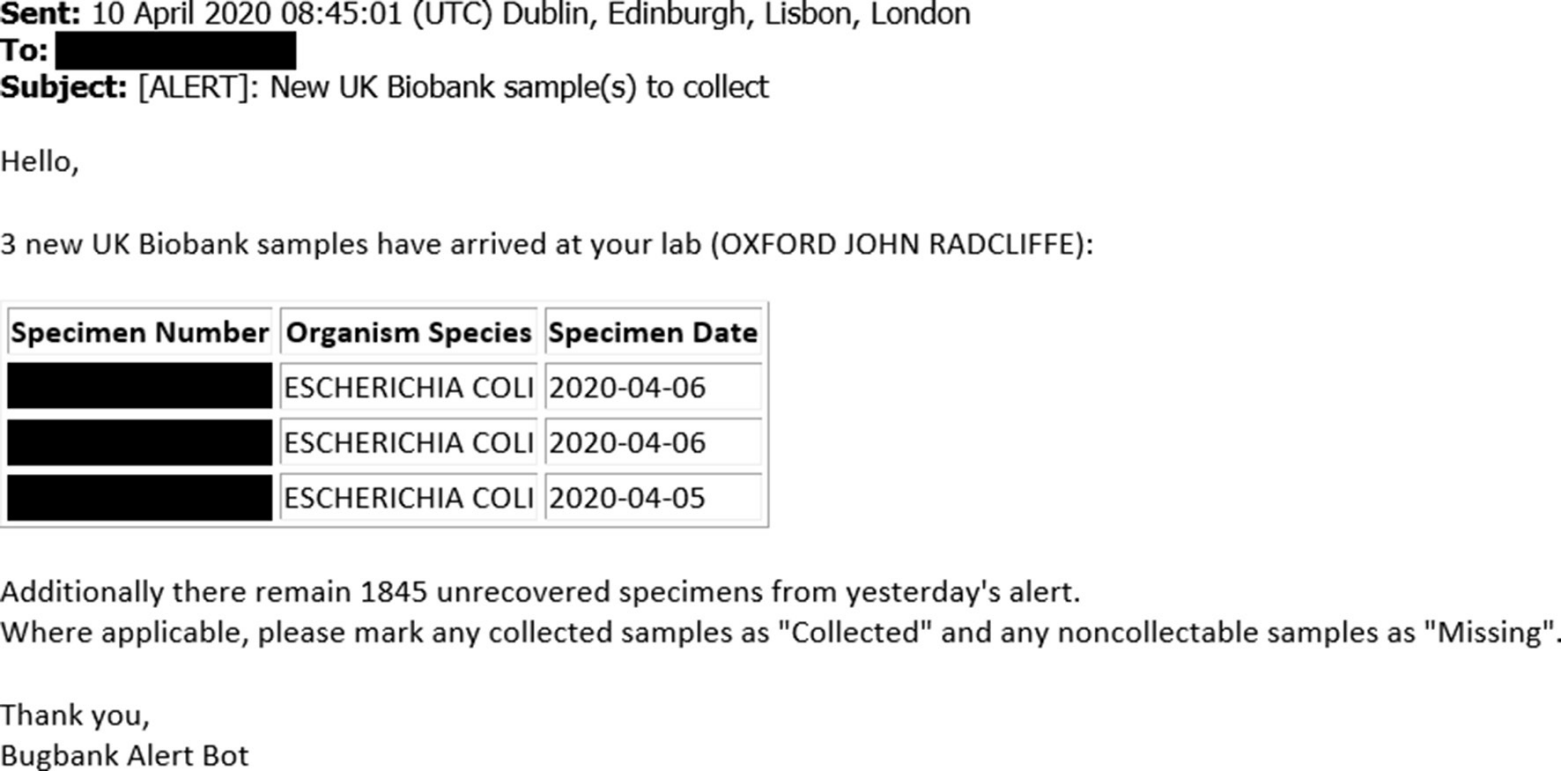
Example email alert to retrieve micro-organisms cultured from UKB participants’ infections. The alert is sent automatically from PHE Colindale to the NHS or PHE laboratory. It contains minimal information necessary to retrieve the micro-organisms.

- (4) The agent sends an email alert to the key person, e.g. a biomedical scientist, at the NHS/PHE laboratory to alert them that new samples have arrived (Fig. 2).

- (5) The key person accesses the details of the microbiological cultures necessary for retrieval, retrieves the identified sample and, if appropriate, makes a stock of the microbial growth for freezer storage. Each sample is assigned a unique sample identifier and storage location that is logged into the secure system. The key person logs any samples that could not be located and other non-personal information relating to the sample relevant to the pilot goals, such as noting physical damage, lack of growth or low growth.

**Fig. 3.**
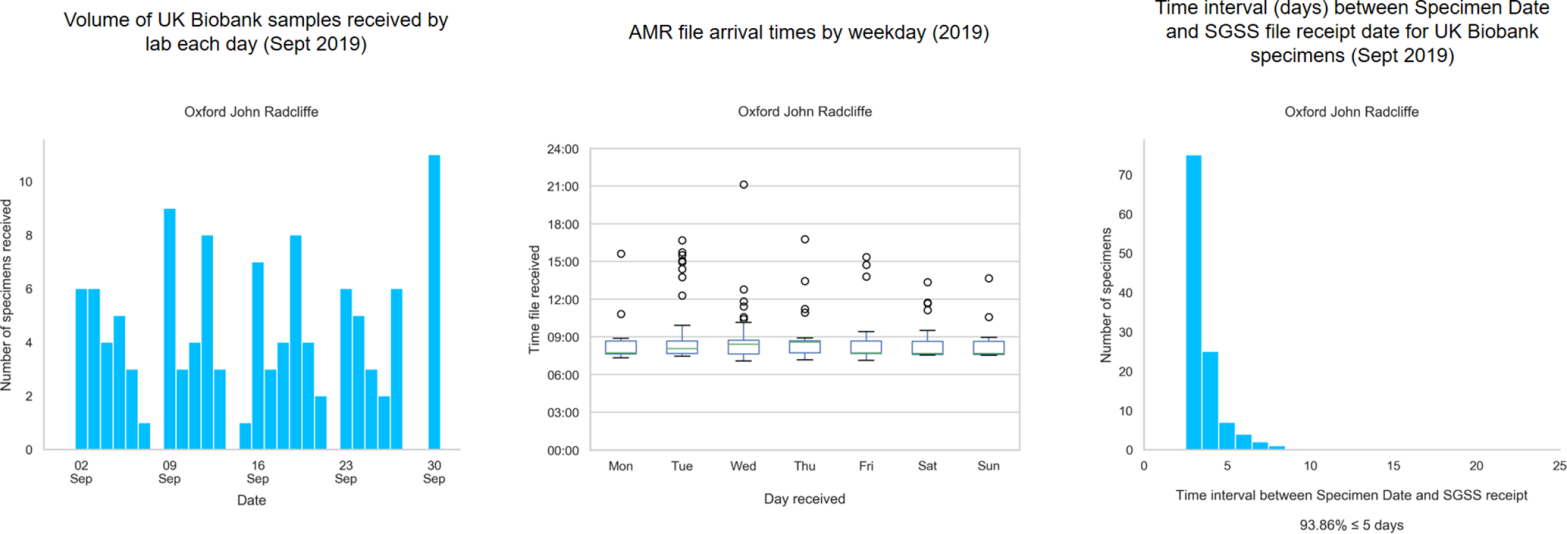
Summary of UKB infection events at the John Radcliffe Hospital, Oxford, during September 2019. The summary indicates the volume of events by date (left), the time of day (middle) and the time to record receipt in SGSS (right).

To test the functionality of the system, we implemented an automated bi-daily SGSS-UKB cohort linkage, with automated daily email alerts of any new records. The prospective pilot study was commenced, with email alerts describing which samples to target sent to the relevant laboratory through the PHE secure network (Fig. 2). We have tested this system by collecting bacteriology samples in the John Radcliffe Hospital, Oxford, prior to proposed England-wide deployment, although it could also be applied to COVID-19 samples. Fig. 3 summarizes the UKB participants’ infection events reported by the microbiology laboratory of the John Radcliffe Hospital, Oxford, in September 2019, which is among the 10 English laboratories with the most-frequent UKB infections. The outcomes of the pilot study, including retrieval rates, will be presented in future work, but initial results indicate that the dynamic record system does allow us to retrieve samples in a timely manner before specimens are discarded, as is routine in microbiology laboratories (14/31 samples in a run-through conducted 24–28 February 2020 at the John Radcliffe Hospital).

### Characterization of the first detected cases of COVID-19 in UKB

#### Working definition for identifying severe COVID-19 cases in UKB

A key question for the international research community is what factors predispose individuals to severe COVID-19? We considered whether these individuals could be identified from the data available. Although SGSS does not contain clinical illness severity (this will come from linking UKB to electronic medical records), SGSS does contain the hospital origin of samples. This is relevant because, from 16 March 2020, the UK entered a suppression phase aimed at delaying the COVID-19 outbreak, during which COVID-19 testing was largely restricted to inpatients, and hospitalization was restricted to those requiring medical support. Indeed, even access to accident and emergency (A and E) departments for patients with suspected COVID-19 requires assessment by a telephone service (111), which only refers severe cases to hospital. In contrast, during the preceding containment phase, referral to hospital was practised even for those with very mild disease for infection control reasons.

Therefore, for individuals sampled from 16 March 2020, we propose that testing positive for SARS-CoV-2 as a hospital inpatient is an appropriate surrogate of severe disease for initial analyses. This definition is not necessarily sensitive, as individuals tested in the community and subsequently admitted are not included in our definition unless they are re-tested in hospital and found positive.

From SGSS, we define a *hospital inpatient* test result as any SARS-CoV-2 test result having one or more of the following properties:

- the Requesting Organization Type associated with the test was either ‘Hospital Inpatient’ or ‘Hospital A and E’,
- the record possessed an ‘Acute Trust’ flag, meaning that the test came from a hospital delivering emergency care,
- or the record possessed a ‘Hospital Acquired Infection’ flag.

Note that this definition excludes testing of individuals because they are a ‘healthcare worker' (i.e. the associated Requesting Organization Type is ‘Healthcare Worker Testing’). For subsequent analyses, we identified SARS-CoV-2 positive UKB participants as hospital inpatients if any positive test result from that individual was defined as a hospital inpatient test result. Manual curation of a sample of records identified by this method, and inspection of free-text information about ward or other sampling locations, indicated that this definition provided a specific means of identifying inpatient samples.

We have linked data at the individual test level and included both positive and negative test results corresponding to English UKB participants. UKB released the first such data tranche on 17 April 2020. In the data released by UKB (data field 40100), SARS-CoV-2 test result is coded in the *result* column as positive (1) or negative (0). Hospital inpatient status, as defined above, is coded in the *origin* column as inpatient (1) or non-inpatient (0). Some of these non-inpatient tests may correspond to individuals subsequently admitted to hospital, and so the non-inpatient designation does not necessarily reflect mild disease.

The *laboratory* column in the data released by UKB records the originating diagnostic laboratory for the test result. Negative test results are processed differently to positives because, for organisms other than SARS-CoV-2, they are not usually recorded in SGSS. Several laboratories reporting many positives do not report negatives directly to SGSS for technical reasons [notably the Northern General Hospital (Sheffield), St George’s Hospital (Tooting), Leeds General Infirmary, John Radcliffe Hospital (Oxford), Darent Valley Hospital (Dartford) and Royal Liverpool University Hospital), while some have reported negatives directly to SGSS intermittently (notably Sunderland Royal Infirmary). Negative results for these laboratories are processed via the Respiratory DataMart, a separate system. This process is imperfect and causes idiosyncrasies for some laboratories in the identification of, and hence the apparent proportion of, negative test results as inpatient versus non-inpatient. Efforts are ongoing to improve the consistency of data reporting, but there are evolving challenges. Further internal data processing changes are expected with moves towards larger-scale testing (coronavirus.data.gov.uk/about). Downstream users should beware of the potential for artefacts when comparing positive and negative test results, unless these idiosyncrasies are accounted for.

Of note, future integration into UKB of HES and Intensive Care Unit (ICU) data that record severity and augmented care periods information (e.g. intensive and high dependency ward stays) is planned in the future. Therefore, more refined classifications of disease severity may be provided in later releases of the UKB data.

#### Demographic characteristics of UKB participants with severe COVID-19

Between 16 March and 19 May 2020, 895 UKB participants reported a positive SARS-CoV-2 test while hospital inpatients in England. A further 457 SARS-CoV-2 positive participants were excluded from analysis: 23 inpatients who tested positive before 16 March, 3 non-inpatients who tested positive before 16 March and 431 non-inpatients who tested positive on or after 16 March.

The total number of PCR-confirmed COVID-19 cases between 16 March and 14 May (allowing a reporting lag of 5 days) in England, reported by the UK Government, was 140 660 (data from https://coronavirus.data.gov.uk). The total number of UKB participants with SARS-CoV-2 positive tests in SGSS over the same period was 1304, of whom we classed 888 as inpatients. Thus, UKB participants meeting our operational definition of severe (i.e. hospitalized) COVID-19 currently comprise 0.6 % of the total number of COVID-19 cases reported in public UK Government data for England, allowing UKB researchers to anticipate changes in sample size from public data released daily.

**Fig. 4.**
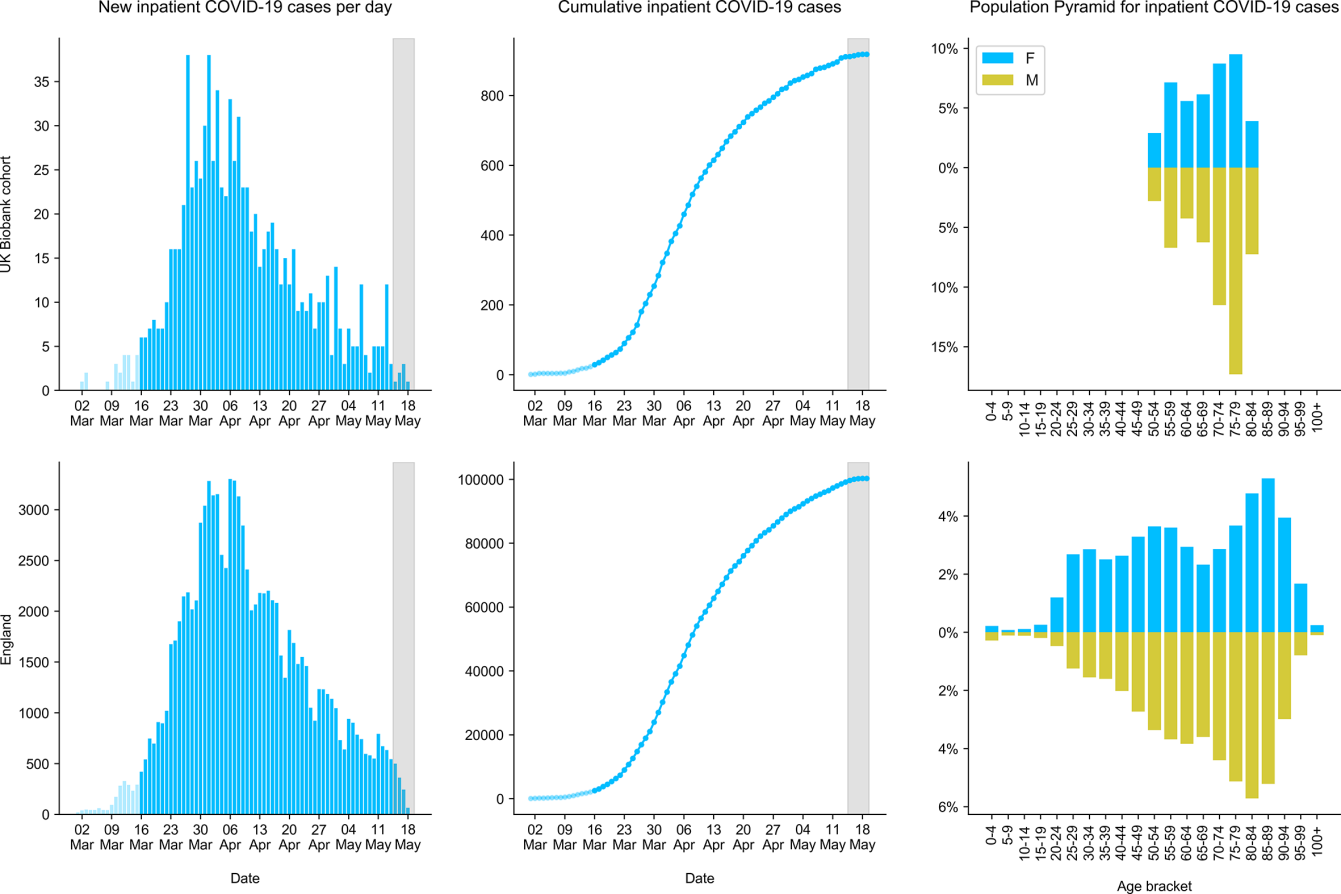
Demographic features of individuals with COVID-19 among English UKB participants (top) and all individuals in the PHE SGSS (bottom). Only hospital inpatients are shown, since these cases can be inferred as severe COVID-19, because only severe cases were admitted to hospital from 16th March 2020 onwards. COVID-19 is determined by positive PCR for SARS-CoV-19. Panels show the total number of new cases per day (left), cumulative number of cases (middle), and the age and sex distribution of cases (right). The dark grey shaded region (left and middle panels) highlights the reporting lag period for some cases, assessed as around 5 days.

The number of new cases of COVID-19 inpatients recorded in SGSS has increased rapidly since early 2020, with close correspondence between the growth in cases among UKB participants and England as a whole (Fig. 4). The total number of inpatients with SARS-CoV-2 positive tests between 16 March and 14 May 2020 in England, recorded by SGSS, was 97 071. Thus, UKB participants made up 0.9 % of all COVID-19 inpatients in SGSS. The change in this portion over time was not significantly different to zero, although there was statistical uncertainty [95 % confidence interval (CI) −1.9–0.6 % per day, Poisson regression]. Thus, the outbreak dynamics appear broadly similar between UKB participants and the general population of England.

**Fig. 5.**
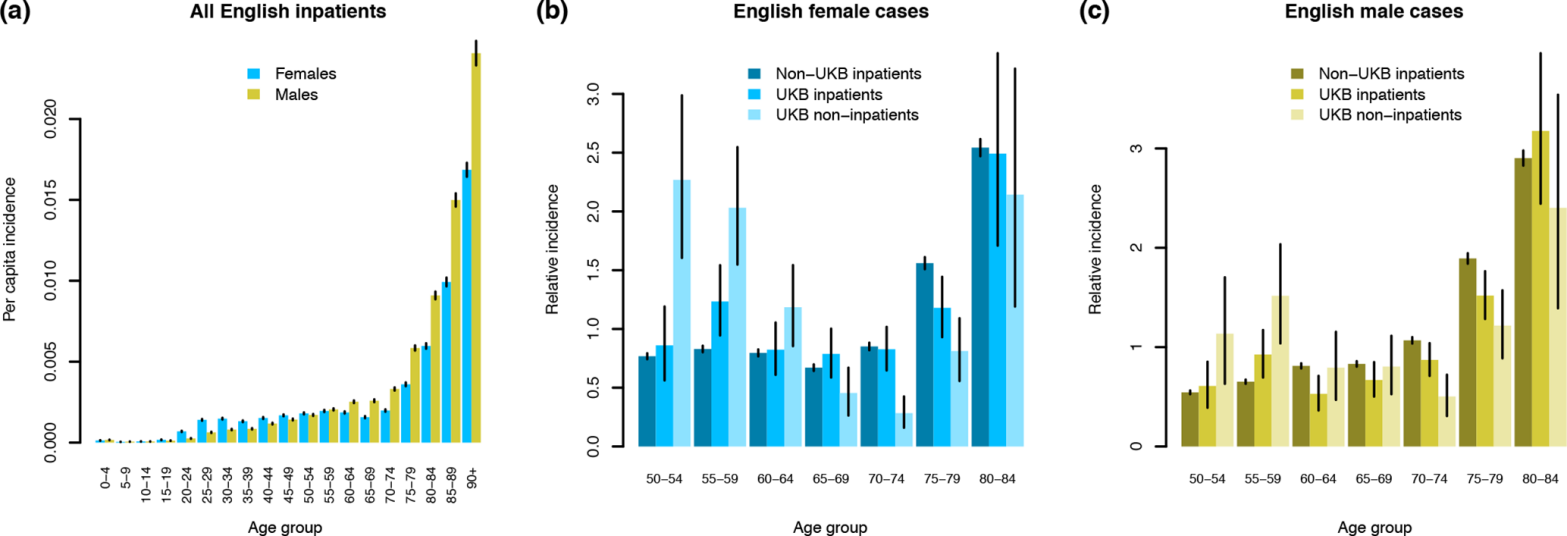
Incidence of SARS-CoV-2 positive individuals in England, 16th March – 19th May 2020. Per capita incidence is show for all English inpatients (a). Relative incidence (per capita incidence in each age group divided by mean per capita incidence across age groups) is compared between non-UKB inpatients, UKB inpatients and UKB non-inpatients for females (b) and males (c). Vertical black lines indicate 95 % CI values calculated assuming a Poisson distribution for the underlying counts. Incidence was calculated using the known age and sex distribution for England as a whole, and English UKB participants.

We estimated per capita incidence of our operational definition of severe COVID-19 for the period 16 March – 14 May 2020 using SGSS data, not restricted to UKB participants (Fig. 5a). In keeping with other reports (see Introduction), males over 50 were generally at elevated risk of developing severe COVID-19 across England (odds ratio 1.41, 95 % CI 1.39–1.43; Cochran–Mantel–Haenszel test stratified by age group), relative to females over 50. We compared UKB positive inpatients to all positive inpatients in England using SGSS data to test for systematic differences in incidence by age and sex. Because of the recruitment strategy used, UKB participants differ from the general population in their age and sex profile [3, 14]. Taking these differences into account using the age and sex distribution of the English UKB cohort in early 2020 and the Office for National Statistics estimates of the English population from mid-2018 (https://www.ons.gov.uk/peoplepopulationandcommunity/populationandmigration/populationestimates/datasets/populationestimatesforukenglandandwalesscotlandandnorthernireland), we compared disease incidence in UKB versus the general population. The absolute number of UKB positive inpatients was 72.6 % (67.9–77.5 %) of the expected total (*P*=1.8×10^−21^; Cochran–Mantel–Haenszel test stratified by age and sex), perhaps indicating that UKB participants are healthier or less exposed on average than the general population [14]. Even taking into account this difference, the relative incidence of severe COVID-19 by age and sex was not identical between non-UKB inpatients and UKB inpatients (*P*=0.005 females, *P*=0.0005 males, chi-square goodness-of-fit tests; Fig. 5b, c). The age distributions of positive UKB participants categorized as inpatients and non-inpatients differed (*P*=1.8×10^−9^ females, *P*=0.001 males), with younger participants (under 65) over-represented among non-inpatients (Fig. 5b, c). In conclusion, incidence of severe COVID-19 by age and sex is similar but not identical between the English UKB cohort and the rest of the population, with 27.4 % fewer positive inpatients than expected.

**Fig. 6.**
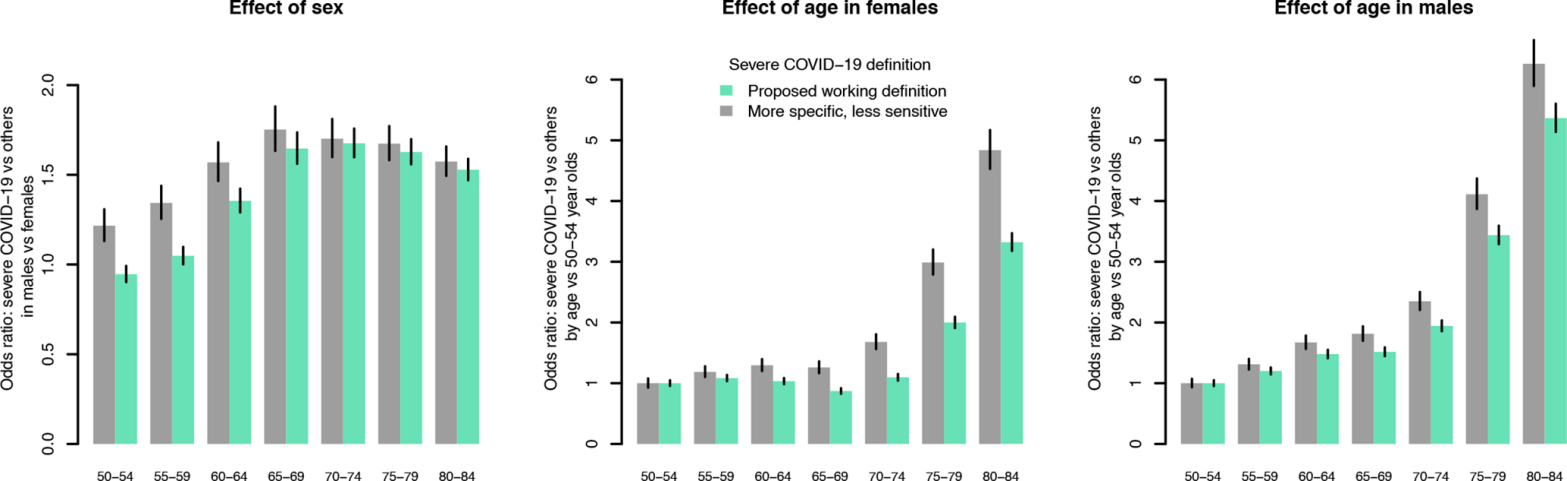
Impact of alternative definitions of severe COVID-19 on estimated effects of age and sex on incidence. PCR-positive hospitalized inpatients are considered to represent severe cases. Two methods of identifying inpatients are compared, the proposed working definition (green) and a more specific but less sensitive method (grey). For each definition, the effects of age and sex on the odds of severe COVID-19 were estimated using fisher.test in R and compared.

We investigated the robustness of our method of identifying hospital inpatients, on which we base the definition of severe COVID-19. We compared the proposed definition, stated above, to an alternative that identifies hospital inpatients as only those with Requesting Organization Type equal to ‘Hospital Inpatient’. This definition is less sensitive, identifying only 509 inpatients compared to 895. Fig. 6 indicates that the alternative definition (grey bars) may be more specific, because the odds ratios of severe COVID-19 were larger (further from 1) than under the proposed definition. We conclude that greater sensitivity of the proposed definition (green bars) trades off some specificity compared to a more stringent alternative and, therefore, modestly dilutes the magnitude of age- and sex-specific differences in severe COVID-19 incidence.

#### Power calculations for genome-wide association studies (GWASs)

If we could predict how numbers of ascertained cases of severe COVID-19 in the UKB cohort will increase over time, we could calculate the power of statistical analyses to discover risk factors. Since it is difficult to predict the outbreak trajectory, we investigated the statistical power to detect human genetic risk factors for severe COVID-19 as a function of the possible number of future cases. This is useful, because the absolute number of new cases in UKB can be roughly estimated as a proportion of the total new cases published daily, assuming current testing trends continue, as detailed above.

We considered the power of a GWAS to detect a rare human allele that increases the risk of severe COVID-19. This is not the only analysis of interest, as UKB contains detailed information on lifestyle and medical variables in addition to human genetics. However, the calculation for a GWAS may be instructive because of its large scale (circa 800 000 directly genotyped variants) and standardized approach. In particular, testing on this scale attracts a highly stringent multiple testing significance threshold of *P* <5×10^−8^, so the GWAS example is a conservative illustration compared to other analyses that do not require such stringent adjustment for multiple comparisons.

**Fig. 7.**
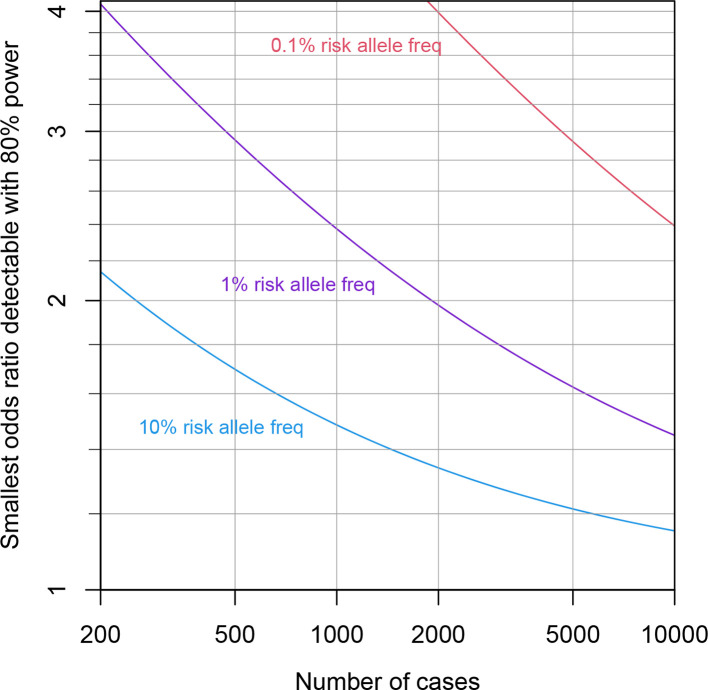
Power calculations for GWASs. The smallest odds ratio (case/control status versus risk/protective allele) detectable with 80 % power is shown as the number of cases increases from 200 to 10 000, assuming a genome-wide significance threshold of 5×10^−8^.

Fig. 7 shows the smallest detectable odds ratio at 80 % power, as a function of sample size and risk-allele frequency. The odds ratio quantifies the relative probability of case versus control status for individuals possessing the risk versus protective allele. We made a range of simplifying assumptions: that the sample frequency of cases is 73 % of the population frequency (see above), that the variant is not on a sex chromosome, that the variant is in Hardy–Weinberg equilibrium, that two copies of the risk allele squares the odds ratio, that the white European subset of circa 350 000 individuals is analysed, that population stratification of the risk allele is negligible and that the risk allele is the causal variant, rather than a linked variant. We calculated the power using the bpower function of the Hmisc package in R (https://cran.r-project.org/package=Hmisc).

The calculations indicate that even with 5000 cases, a number we consider high given the current outbreak trajectory in England, the above analysis would have high (80%) power to detect only relatively large odds ratios exceeding 1.2 for risk alleles at 10 % population frequency. For rarer risk alleles (1 and 0.1%), the odds ratios detectable with 80 % power increase to 1.6 and 2.9, respectively. While odds ratios of these magnitudes are known for some infection susceptibility variants, many known variants possess more modest odds ratios below 1.2 [15, 16].

Our calculations do not take into consideration boosts in power that can be achieved by various means, including pooling the effects of multiple variants within or between genes using analyses of various kinds [17] or meta-analysis of the sort planned by the COVID-19 Host Genetics Initiative (www.covid19hg.org), which aims to combine signals across multiple cohorts.

## Discussion

There are several limitations to this work. We rely on microbiological testing, which has been largely restricted to hospitalized cases. Under-ascertainment of severe COVID-19 in community settings, for example nursing homes, is therefore highly likely. Even where SARS-CoV-2 PCR tests have been performed, we cannot assume that the assay is fully sensitive. Since COVID-19 severity scores are not yet readily available, we have made the assumption that hospitalized cases with SARS-CoV-2 positive tests are a proxy for severe COVID-19. The method by which hospital inpatients are identified may affect downstream analyses, and currently possesses some idiosyncrasies for negative test results. In the analyses presented here, we have not distinguished those individuals with only positive tests from those with a mixture of positive and negative tests. Integration of further data sources may mitigate some of these limitations, adding information on clinical disease severity and admission to intensive care, which is collected for some individuals in the PHE CHESS and ICNARC databases.

We are unable to assess exposure to SARS-CoV-2 in most UKB participants. This has important implications for case–control studies, because we cannot distinguish individuals who have not contracted SARS-CoV-2 following exposure from those who have not been exposed. As the outbreak progresses, exposure and cases of severe COVID-19 will increase. Any case–control definition is, thus, inherently dynamic, and this will affect analysis and interpretation. Moreover, the nature of the SGSS resource and future changes in national testing mean that interpretation of the data feed remains fluid; we will review such changes and provide updates via the project website (www.bugbank.uk).

Despite its limitations, the linkage of COVID-19 test results to the UKB provides a valuable resource to the international research community that has the potential to uncover new risk factors for severe infection. UKB is one of the largest and closest-studied cohorts in the world. A wide range of detailed epidemiological risk factors encompassing lifestyle and medical variables are available to UKB-registered researchers to study, in addition to human genotyping and a variety of other technologies, such as exome sequencing in some participants. Beyond UKB, we are applying our dynamic linkage system to facilitate linkage of COVID-19 PCR positive and negative test results to other UK cohort studies, including INTERVAL, COMPARE (www.donorhealth-btru.nihr.ac.uk), Genes & Health (www.genesandhealth.org) and the NIHR BioResource (bioresource.nihr.ac.uk). Collaboration with these other cohorts increases the potential value of this work to the effort to understand and tackle COVID-19.

Our work has the potential for wider impact beyond enabling urgent research into COVID-19, because it makes it possible to prospectively sample microbiological cultures from UKB participants that will – subject to detailed assessment through an ongoing culture collection feasibility study – afford an opportunity to study microbiological and molecular genetic risk factors for a range of other important pathogens.
